# Addressing non-communicable disease management during disasters in the US Virgin Islands: a mixed methods study

**DOI:** 10.3389/fpubh.2025.1606631

**Published:** 2025-09-12

**Authors:** Michelle Teresa Wiciak, Stephen Perez, Tess Richards, Karla Escobar, Dabney P. Evans, Marcella Nunez-Smith, Saria Hassan

**Affiliations:** ^1^Department of Medicine, Emory University School of Medicine, Atlanta, GA, United States; ^2^Hubert Department of Global Health, Emory Rollins School of Public Health, Atlanta, GA, United States; ^3^St Thomas East End Medical Center Corporation, St Thomas, US Virgin Islands; ^4^Department of Medicine, Yale University School of Medicine, New Haven, CT, United States

**Keywords:** climate change, non-communicable diseases, US virgin islands, resilience, disasters, preparedness

## Abstract

**Introduction:**

Extreme weather events, like hurricanes Irma and Maria, have disproportionately impacted individuals with non-communicable diseases (NCDs) in the U.S. Virgin Islands (USVI), exacerbating health disparities due to healthcare disruptions. The USVI and other Caribbean islands face increased morbidity and mortality from NCDs from rising risk factors and lack of improving in quality of care. This study explores the experiences of individuals with NCDs during these hurricanes to identify strategies for improving disaster preparedness and response.

**Methods:**

A mixed-methods cross-sectional study was conducted at a Federally Qualified Health Center (FQHC) in St. Thomas, USVI. One-hundred and thirteen quantitative surveys assessed preparedness, healthcare access, and mental health impacts. Fifteen semi-structured qualitative interviews provided deeper insights into patient experiences and coping strategies. Data integration followed a narrative approach.

**Results:**

Quantitative findings revealed nearly one third of participants struggled to manage their NCDs post-disaster, citing stress-related exacerbation of conditions (42.3%), lack of medication access (34.6%), and disrupted healthcare services (34.6%). Mental health burdens were significant, with nearly a third reporting anxiety (28%) and depression (27.8%), and 5.2% meeting PTSD criteria. Many participants (39.7%) had not received disaster preparedness information tailored to NCDs, with only 47.5% receiving guidance from healthcare providers. Qualitative interviews underscored these findings, highlighting that NCD management was deprioritized post-disaster due to immediate survival needs. Participants emphasized the role of family and community support in coping, yet also noted mental health stigma and limited healthcare access as ongoing barriers. Preferred communication strategies included social media, radio, and trusted sources.

**Conclusion:**

Findings reveal critical gaps in disaster preparedness for persons with NCDs in the USVI. Strengthening healthcare infrastructure, enhancing mental health support, and providing targeted education can improve resilience and reduce morbidity in future disasters.

## 1 Introduction

Globally, disasters are becoming more deadly and costly due to their increased frequency and intensity. In 2024 alone, it was estimated that there were 167.2 million people impacted by disasters, with 16,753 deaths, and 320 billion US dollars in overall losses (1). The most recent report of the Intergovernmental Panel on Climate Change (IPCC) indicated that, as a result of climate change, the world will continue to see an increase in the frequency of severe storms (2). Data from the World Health Organization (WHO) highlight that an estimated 3.6 billion people currently live in areas highly susceptible to climate change. The Caribbean region is considered to be “ground zero” for the climate crisis; island nations and states in the Caribbean face the compound stressors of extreme heat, increased rainfall, severe hurricanes, and sea level rise (3). Damages due to climate change in the Caribbean are expected to cause an increase in costs from 5% of gross domestic product (GDP) in 2025 to more than 20% by 2,100. Much of this cost is due to the significant multi-level impact of hurricanes (4).

In 2017, two Category 5 storms devastated the Caribbean US territories within 2 weeks of each other. Hurricane Irma directly hit St. Thomas and St. John on September 6, 2017, and Hurricane Maria directly hit St. Croix and Puerto Rico on September 20. The hurricanes resulted in utter and complete destruction and devastation of infrastructure on both US territories (5, 6). In Puerto Rico, the official death toll of 64 was later revised to greater than 4,000 after accounting for excess mortality (7). In fact, studies on the mainland US indicated that at least 30% of deaths after Hurricane Irma were due to poorly managed non-communicable diseases (NCDs), which was likely the case in Puerto Rico (5, 8). Unfortunately, limited data availability has hindered such studies in the US Virgin Islands (USVI) of St. Thomas, St. Croix, and St. John; there are no estimates on excess mortality to more accurately and reliably reflect the true death toll. This makes it difficult to objectively assess the depth of the damage and, importantly, to inform future disaster preparedness and response.

Global disasters have impacted patients living with NCDs in a myriad of ways. In the aftermath of a 2008 earthquake in Sichuan, China, researchers learned that patients living with chronic health needs required additional medication and represented a majority of those requiring emergency care immediately after the storm (9). A systematic review of the impacts of disasters on NCDs found that diabetes patients’ disruption in medical care, lack of insulin and access to medication can compound their treatment and ultimately exacerbate their disease (10). Furthermore, this review highlighted how worsening symptoms like shortness of breath for those living with chronic respiratory disease can result in the aftermath of disasters. For those living with mental health disorders, worsening symptoms, the sudden change in the acute phase of a disaster, and shortage of medications can impact patients’ ability to maintain their own mental wellbeing. Another scoping review of NCDs in the setting of hurricanes in the Caribbean highlighted the need to address access to medication and access to healthy food, the need for mental health services, and general chronic disease management (11). Ultimately, floods, earthquakes, hurricanes, and other disasters impact those living with NCDs in a range of complex and challenging ways in the immediate and long-term aftermath of these disasters (10).

The impact of hurricanes on NCDs is especially problematic in the Caribbean which, at baseline, has the highest premature mortality from NCDs in the all the Caribbean (12). The US territory of the US Virgin Islands similarly suffers from a high burden of NCDs and NCD related mortality. A Pan American Health Organization (PAHO) study found that of the 22 Caribbean countries/territories investigated from 1999 to 2014 looking at cumulative 10-year proportions of death from 4 NCDs, the USVI had the highest percentage of deaths due to heart disease (27%) (13). Combined with the other three NCDs (Cancer, cerebrovascular disease, and diabetes), the USVI had the 7^th^ highest cumulative proportions of deaths due to all four NCDs, with 57% of deaths due to these four NCDs (13). The high prevalence of NCDs in the Caribbean including the USVI is the result of a high burden of risk factors including an unhealthy diet with high consumption of sugar-sweetened beverages (14), low fruit and vegetable consumption (15), and high rates of food insecurity (16); high prevalence of obesity at above 30% in the USVI in 2023 (17); and high alcohol use (18). It is estimated that 80% of adults have at least one major risk factor for heart disease (19). The USVI also has an aging population, which contributes to rising rates of NCDs; the percentage of adults aged 65 or older rose from 13.5% in 2010 to 21.3% in 2020, according to the 2022 US census (18, 20, 21). In addition to the increasing influence of risk factors, the US territories have been challenged in quality care delivery with significant gaps in 30-day hospital mortality and diabetes management compared to the US mainland (22, 23). This is due to multiple factors, including regulations related to US territories that lead to differential healthcare reimbursement rates and legal restrictions in shaping US policy (24, 25). Repeated exposure to severe hurricanes that destroy healthcare infrastructure also challenges the delivery of continued quality care. The combination of a high burden of NCDs and its location in the Caribbean makes the USVI highly vulnerable to the impact of climate-related extreme weather events on the population’s health (13, 26–28).

The vulnerability of human health to climate change and climate-related disasters can be considered a function of exposure, sensitivity, and adaptive capacity (29, 30). The USVI, situated in the Caribbean, is projected to see a higher frequency and intensity of severe weather events because of climate change. Persons living with NCDs in the USVI have a higher sensitivity and, therefore, vulnerability to these climate-related events. Prior studies investigating the increased susceptibility of persons living with NCDs to disasters have identified contributing factors that include limited access to healthcare services, access to medication, access to healthy food, and mental health stressors (11). This underlying sensitivity is often exacerbated by social determinants of health (SDOH) that significantly worsen outcomes: lack of insurance, low income, lower level of education (including low health literacy), disabilities, and food and housing insecurity. The cumulative effect of high and frequent exposure to climate disasters, underlying sensitivities due to NCDs, and SDOH makes communities in the US territories, including the USVI, highly vulnerable to the disastrous impact of climate change and climate-related disasters on their health. To counteract this heightened vulnerability, we must identify strategies to strengthen their adaptive capacity–the ability to adapt–to the impact of climate-related disasters on persons living with NCDs in the USVI.

In order to strengthen adaptive capacity to disasters among persons living with NCDs, we need to fill a significant gap in our understanding of the barriers and facilitators to NCD management during disasters in the USVI. This paper addresses this gap by exploring the experiences of persons living with NCDs during hurricanes Irma and Maria in the USVI and providing recommendations for strengthening adaptive capacity for improved disaster response in the future.

## 2 Methods

### 2.1 Study design and setting

This study used a concurrent mixed-methods cross-sectional study design with the weaving approach for data integration of the quantitative and qualitative data. This methodology was picked and deemed ideal for this study since mixed methodology is a superior design to provide a robust understanding of the experience of persons living with NCDs faced during the hurricanes (31). This methodological approach helped quantify the challenges faced and also explained the underlying causes, existing barriers, and potential future solutions. Study participants were recruited from a Federally Qualified Health Center (FQHC) on the USVI island of St. Thomas, which was severely affected by the 2017 Hurricanes Irma and Maria.

### 2.2 Recruitment, eligibility, and demographics

For both components of the study, eligible participants included existing patients at the FQHC with an existing NCD diagnosis previously made by a healthcare provider, who had previously lived through a climate-related extreme weather event, were English or Spanish-speaking, could consent, and complete a survey administered through REDCap, on an iPad, or on paper. NCD diagnoses included were high blood pressure, diabetes, high cholesterol, mental health disorders, cardiovascular disorders, respiratory disorders, rheumatoid arthritis, pre-diabetes/borderline diabetes, kidney disease, Hepatitis C, other liver disease, HIV/AIDS, cancer, Systemic Lupus Erythematosus (SLE), and epilepsy (listed in Table 1). Patients were compensated $10 via a Visa reward gift card upon completing the survey and $25 via a Visa reward gift card upon completing the qualitative interview.

**Table 1:** Patient characteristics and other frequencies.

Variable	*N* (%)
Demographics	
Age (*n* = 80)	51.78 (sd = 16.51)
Sex (*n* = 88)	
Male	15 (17.0%)
Female	73 (83.0%)
Education level (*n* = 86)	
Less than a high school degree	11 (12.8%)
High school degree	52 (60.5%)
More than a high school degree (I.e., college, professional)	23 (25.7%)
Has insurance (*n* = 85)	75 (88.2%)
Type of insurance (*n* = 72)	
Medicaid	31 (43.1%)
Medicare	15 (20.8%)
Private	19 (26.4%)
Other	7 (9.7%)
Chronic condition	
Diagnosed chronic health condition (*n* = 113)	
High blood pressure	62 (54.9%)
Diabetes	25 (22.1%)
High cholesterol	24 (21.2%)
Mental health disorders	22 (19.4%)
Cardiovascular disorders	18 (15.9%)
Respiratory disorders	21 (18.6%)
Rheumatoid arthritis	15 (13.3%)
Pre-diabetes/Borderline diabetes	9 (8.0%)
Other*	11 (9.9%)
Place to go when sick/need health advice (*n* = 89)	
No place	5 (5.6%)
One place	51 (57.3%)
Multiple places	33 (37.1%)
Number of times saw health care professional for NCD in past 12 months (*n* = 87)	
Never	8 (9.2%)
1–2 times	32 (36.8%)
3–4 times	34 (39.1%)
4 + times	13 (14.9%)
Usual source of care (*n* = 84)	
None	2 (2.4%)
Clinic/healthcare center	51 (60.7%)
Doctor’s office/HMO	19 (22.6%)
Emergency room	12 (14.3%)
On medication (*n* = 89)	68 (76.4%)
On medication needing refrigeration (*n* = 67)	11 (16.4%)
Number of diagnosed NCDs (*n* = 113)	
1	56 (49.6%)
2	30 (26.5%)
3 or more	27 (23.9%)
Disaster	
Lived through a disaster (*n* = 113)	113 (100%)
Displacement from home during disaster (*n* = 92)	25 (27.2%)
Experienced challenges managing chronic condition (*n* = 88)	26 (29.9%)
Time frame after the disaster that people experienced challenges with their NCD (*n* = 26)	
Within 1 week	9 (34.6%)
1–2 weeks	5 (19.2%)
2–8 weeks	8 (30.8%)
2–3 months	2 (7.7%)
4–6 months	1 (3.8%)
Top conditions that experienced challenges (*n* = 26)	
Diabetes	5 (19.2%)
Rheumatoid arthritis	5 (19.2%)
Asthma	4 (15.4%)
High blood pressure	3 (11.5%)
Top reasons for challenges (*n* = 26)	
Increased stress/anxiety	11 (42.3%)
No access to medication	9 (34.6%)
No access to healthcare	9 (34.6%)
No access to healthy food	7 (26.9%)
% with battery-operated radio currently (*n* = 68)	64 (94.1%)
% who received information on preparing for a disaster (past 6 months) (*n* = 87)	64 (73.6%)
% receiving information specific to chronic disease emergency preparedness in past 6 months (*n* = 58)	35 (60.3%)
% getting information from healthcare providers on emergency preparedness (*n* = 61)	39 (47.5%)
Level of preparedness to manage chronic disease after a disaster (*n* = 82)	
Not prepared	2 (2.4%)
Only a little prepared	4 (4.9%)
Somewhat prepared	26 (31.7%)
Very prepared	31 (37.8%)
Extremely well prepared	19 (23.2%)
Money set aside for emergency (*n* = 80)	39 (48.8%)
Enough supplies for 5 days (*n* = 79)	57 (72.2%)
Mental Health	
PTSD Score (PCL) (*n* = 78)	9.63 (sd = 10.57)
% meets criteria for PTSD from the disaster (cut-off at 31) (*n* = 76)	4 (3.5%)
Anxiety Score (GAD) (*n* = 75)	3.15 (sd = 4.71)
Level of anxiety (*n* = 75)	
Minimal/None	54 (72.0%)
Mild	15 (20.0%)
Moderate/Severe	6 (8.0%)
Depression Score (PHQ) (*n* = 72)	3.25 (sd = 4.06)
Level of depression (*n* = 72)	
None	52 (72.2%)
Mild	14 (19.4%)
Moderate	3 (4.2%)
Moderately severe	3 (4.2%)
% of patients who either were diagnosed with a mental health condition and/or had presence of MH symptoms [anxiety, depression, trauma from the disaster (*n* = 114)]	36 (31.6%)

^*^Other NCDs include: Kidney disease, Hepatitis C, Other liver disease, HIV/AIDS, Cancer, Systemic Lupus Erythematosus (SLE), and Epilepsy.

A convenience sampling method was utilized to recruit eligible patients. Participants were recruited either in the FQHC waiting rooms in July and August 2022 by a research team member or through clinicians disseminating a flyer with a QR code from August to December 2022 (nearly 5 years after Hurricanes Irma and Maria impacted the USVI).

Follow-up one-time qualitative interviews were conducted with a cohort of participants who completed the survey and agreed to a follow-up interview. Participants who completed the survey and agreed to an interview were selectively invited to maximize diversity in age, sex, and type of chronic disease. Participants who did not complete the survey were not eligible to participate in the interview process.

### 2.3 Methods

#### 2.3.1 Quantitative methods

Patient surveys were developed and administered online through Emory University’s REDCap database, available in both English and Spanish. Our community and scientific advisory group reviewed and piloted the survey.

##### 2.3.1.1 Dependent variables

The main outcome variables of interest were mental health status, level of preparedness, and challenge of managing NCD during the disaster.

The mental health section assessed anxiety, depression, and trauma. For trauma, the 20-item self-reported, validated Post-Traumatic Stress Disorder (PTSD) Checklist for the DSM-5 (PCL-5) was used (32). It was modified to ask participants about PTSD symptoms they experienced in the past month due to the disaster. Scores were dichotomized based on the cutoff score. Scores less than 31 were grouped as “unlikely to meet PTSD criteria” and scores 31 and greater were grouped as “likely to meet PTSD criteria” (32).

To assess anxiety, the Generalized Anxiety Disorder 7 (GAD7) was used. The GAD-7 is a 7-item questionnaire assessing self-reported symptoms of anxiety over the past 2 weeks; scores 0–4 suggest “minimal anxiety,” scores 5–9 suggest “mild anxiety,” scores 10–14 suggest “moderate anxiety,” and scores over 15 suggest “severe anxiety” (33).

To assess depression, the Patient Health Questionnaire 9 (PHQ-9) was used. The PHQ-9 is a 9-item questionnaire that measures the severity of depression over the past 2 weeks; scores 0–4 suggesting “none to minimal depression,” scores 5–9 suggesting “mild depression,” scores 10–14 suggesting “moderate depression,” scores 15–19 suggesting “moderately severe depression,” and scores 20–27 suggesting “severe depression” (34). We also grouped the sample as those “having a mental health condition” (anxiety, depression, and/or trauma present based on cut-off criteria) and those not having a mental health condition (anxiety, depression, and/or trauma absent based on cut-off criteria).

###### 2.3.1.1.1 Level of preparedness

Level of preparedness was assessed as a response to the question: “How prepared did you feel before the hurricane?” Low preparedness was defined as “not prepared,” “only a little prepared,” and “somewhat prepared.” High preparedness was defined as “very prepared” and “extremely well prepared.” This dichotomization was based upon percentiles to ensure adequate sample size between the two groups.

###### 2.3.1.1.2 Challenge managing NCD

Challenge managing NCD was determined by response to the question: “In the aftermath of the disaster, did you have any challenges managing your chronic disease(s)?” Response options included: “yes,” “no,” “not sure,” and “decline to say.” If participants answered “yes,” they were then instructed to specify which type of barrier they faced.

##### 2.3.1.2 Independent variables

Sociodemographic information (age, sex, insurance, education level), characterization of type of chronic disease, medications, usual source of care, trauma from disaster, and primary sources of disaster preparedness information were included as independent variables.

##### 2.3.1.3 Analysis

Survey analysis was done using SPSS version 27.0 (IBM Corp, Armonk, NY). All statistics were conducted at the 95% confidence level with a significance level set at alpha = 0.05. Data analysis included descriptive statistics, Fisher’s exact tests, Chi-square analyses, Levene’s test for equality of variances, and independent student t-tests. We removed missing data from the overall data analysis and those who answered “do not know” and “refuse to answer.”

#### 2.3.2 Qualitative methods

Interviews focused on the following topic areas: patient experience with the most recent disasters, preparation, getting help to manage chronic disease during disasters, and lessons learned from past experiences. Interviews were conducted by a trained research assistant (SP), who is a self-identified male and resided in the USVI for 2 years. SP had significant experience and training in qualitative research methodology, specifically conducting interviews, focus groups, and qualitative analysis. Participants knew limited information about the researcher outside of research-related details and no characteristics were reported. Interviews lasted approximately 30–60 min and were conducted virtually via Zoom or over the phone. Only researchers and participants were present for interviews. Sessions were audio recorded with the option for patients to opt-out of being recorded. Researchers conducting the interviews kept field notes. Transcripts were reviewed for accuracy, de-identified, and stored on a secured database only study team members could access. Transcripts were not returned to participants for comment or correction.

Transcripts were analyzed using thematic content analysis as defined by Braun and Clarke (35). First, SP read through transcripts to gain an understanding of some of the areas of focus emerging in the data. A preliminary codebook was then developed based on the interview guide and initial patterns in the data. Team members used the guide as they individually coded transcripts to refine the codebook. Team members met and agreed on early versions of codebooks; after 5 transcripts were individually coded by team members, the team finalized the codebook. The team then used that finalized codebook to code all transcripts. Groups of 2 to 3 team members coded each transcript and met to discuss any discrepancies following the application of codes. After all transcripts were coded, they were re-coded using Dedoose qualitative software for analytic purposes. Themes were generated as the data was compared within and across interviews. Emergent themes were discussed and agreed upon as coded transcripts were reviewed. Themes and sub-themes were refined as the data was considered in accordance with the purpose of our data collection. Thematic saturation was reached with the analysis of completed interviews. Participants did not provide feedback on findings. This qualitative analysis follows guidelines established by the Consolidated Criteria for Reporting Qualitative Health Research (COREQ).

### 2.4 Data integration

A narrative approach is used to integrate quantitative and qualitative data. Quantitative and qualitative data were analyzed separately. The main quantitative and qualitative findings were woven together (weaving approach) to draw a deeper understanding of the challenges and potential solutions to addressing NCD needs in a disaster in the USVI.

### 2.5 Ethical considerations

The Emory University Institutional Review Board reviewed and approved this study. All study participants were appropriately consented in English or Spanish. Participants were allowed to stop the study and withdraw at any time.

## 3 Results

### 3.1 Quantitative results

A total of 113 participants completed the survey; no participants dropped out. Eleven and a half percent (*n* = 13) of participants completed the survey in Spanish, and 88.5% (*n* = 100) completed it in English. A summary of demographic findings is provided in Table 1. Participants had an average age of 51.78 years (SD 16.41), 83% were female, and almost 90% had insurance. The most common NCD was high blood pressure (54.9%), followed by diabetes (22.1%), high cholesterol (21.2%), and mental health disorders (19.4%) with over 50% of participants having two or more NCDs. Slightly over three-fourths (76.4%) were taking medication for their NCD, with 16.4% of those requiring refrigeration.

All participants experienced a disaster, and slightly over one-fourth (27.2%) were displaced from their homes during the most recent disaster. Out of all the NCDs collected in this project, patients with diabetes (19.2%) and rheumatoid arthritis patients (19.2%) experienced the most challenges managing their NCD during the disaster, with asthma (15.4%) and hypertensive patients (11.5%) following. Over 40% of those who experienced challenges said that their reason for the challenge was increased stress and anxiety. In addition, patients mentioned that no access to medication (34.6%) and no access to healthcare (34.6%) were other challenges.

Nearly 40% of patients had a low level of preparedness to manage their NCDs after a disaster. Only 60.3% of participants received information related to emergency preparedness and their NCD in the past 6 months and slightly under 50% said they received information from their healthcare provider on emergency preparedness. A little more than 50% did not have money set aside for emergencies, 28% did not have enough supplies for 5 days, and 43% did not have a battery-operated radio. Most participants received their information on disaster preparedness through the television (61.1%), internet (44.4%), and conversing with others (40.0%). The most trustworthy sources to receive information on disaster preparedness were family (56.7%), government (54.4%), and the media (52.2%).

The average PCL-5 score was 9.49 (sd = 10.58), with 5.2% of participants meeting the criteria for PTSD from the disaster (Table 1). The average GAD-7 score currently for participants was 3.15 (sd = 4.71), with 28% of participants having some level of anxiety (Table 1). The average PHQ-9 score was 3.25 (sd = 4.06), with nearly 28% of participants having some level of depression (Table 1).

It was found that sex (*p* = 0.033), number of NCDs (*p* = 0.028), using medication for NCDs (*p* = 0.044), place to go for care (*p* = 0.023), number of times saw a healthcare professional for NCD in the past 12 month (*p* = 0.002), depression (*p* = 0.002), anxiety (*p* = 0.002), and displacement from the disaster (*p* = 0.035) were associated with whether the patient experienced challenges managing their NCD during the disaster (Table 2). Having a usual source of care (*p* = 0.041) and depression (*p* = 0.042) were associated with the reported level of preparedness (high vs. low) as shown in Table 2.

**Table 2:** Associations found with challenges and preparedness.

Variable	Challenges experienced managing NCDs during disaster			Preparedness		
	Did not experience challenge(s) managing NCD during disaster (*n* = 61) n (%)/x(sd)	Experienced challenge(s) managing NCD during disaster (*n* = 26) n(%)/x(sd)	*p*-value	Not/Only/Somewhat prepared (Low) (*n* = 32) n (%)/x(sd)	Extremely well/Very prepared (High) (*n* = 50) n (%)/x(sd)	*p*-value
Age						
	53.77 (sd = 16.72)	46.63 (sd = 16.05)	*p* = 0.083	51.81 (sd = 18.08)	51.68 (sd = 15.68)	*p* = 0.487
Sex						
Male	14 (23.7%)	1 (4.0%)	*p* = 0.033*	4 (12.5%)	10 (20.4%)	*p* = 0.357
Female	45 (76.3%)	24 (96.0%)		28 (87.5%)	39 (79.6%)	
Insurance						
No	4 (7.0%)	5 (20.8%)	*p* = 0.071	4 (12.5%)	5 (10.6%)	*p* = 0.798
Yes	53 (93.0%)	19 (79.2%)		28 (87.5%)	42 (89.4%)	
Education Level						
Less than a high school degree	6 (10.3%)	5 (20.8%)	*p* = 0.220	5 (16.1%)	5 (10.2%)	*p* = 0.241
High school degree/equivalent	38 (65.5%)	11 (45.8%)		15 (48.4%)	33 (67.3%)	
More than a high school degree	14 (24.1%)	8 (33.3%)		11 (35.5%)	11 (22.4%)	
Number of chronic diseases						
1	32 (52.5%)	7 (26.9%)	*p* = 0.028*	12 (37.5%)	25 (50.0%)	*p* = 0.267
2+	29 (47.5%)	19 (73.1%)		20 (62.5%)	25 (50.0%)	
Using medication for NCD(s)						
No	17 (27.9%)	2 (8.0%)	*p* = 0.044*	6 (18.8%)	11 (22.4%)	*p* = 0.689
Yes	44 (72.1%)	23 (92.0%)		26 (81.3%)	38 (77.6%)	
Medication requiring refrigeration						
No	34 (77.3%)	21 (95.5%)	*p* = 0.062	23 (88.5%)	32 (84.2%)	*p* = 0.631
Yes	10 (22.7%)	1 (4.5%)		3 (11.5%)	6 (15.8%)	
Place to go when sick/need health advice						
No place	2 (3.4%)	3 (11.5%)	*p* = 0.023*	2 (6.5%)	3 (6.1%)	*p* = 0.709
One place	40 (67.8%)	10 (38.5%)		16 (51.6%)	30 (61.2%)	
Multiple places	17 (28.8%)	13 (50.0%)		13 (41.9%)	16 (32.7%)	
Usual source of care						
None	1 (1.8%)	1 (4.3%)	*p* = 0.636	1 (3.4%)	1 (2.2%)	*p* = 0.041*
Clinic/healthcare center	37 (64.9%)	14 (60.9%)		23 (79.3%)	24 (52.2%)	
Doctor’s office/HMO	13 (22.8%)	4 (17.4%)		2 (6.9%)	14 (30.4%)	
Emergency room	5 (10.5%)	4 (17.4%)		3 (10.3%)	7 (15.2%)	
Number of times saw health care professional for NCD in past 12 months						
Never	6 (10.3%)	1 (4.0%)	*p* = 0.002**	3 (9.7%)	5 (10.6%)	*p* = 0.483
1–2 times	24 (41.4%)	6 (24.0%)		8 (25.8%)	18 (38.3%)	
3–4 times	25 (43.1%)	8 (32.0%)		13 (41.9%)	19 (40.4%)	
4 + times	3 (5.2%)	10 (40.0%)		7 (22.6%)	5 (10.6%)	
Trauma from disaster						
Trauma unlikely	48 (96.0%)	23 (92.0%)	*p* = 0.467	28 (93.3%)	41 (95.3%)	*p* = 0.710
Trauma probable	2 (4.0%)	2 (8.0%)		2 (6.7%)	2 (4.7%)	
Depression						
Depression absent	41 (83.7%)	11 (47.8%)	*p* = 0.002**	16 (57.1%)	32 (80.0%)	*p* = 0.042*
Depression present	8 (16.3%)	12 (52.2%)		12 (42.9%)	8 (20.0%)	
Anxiety						
Anxiety absent	43 (84.3%)	11 (50.0%)	*p* = 0.002**	17 (65.4%)	33 (76.7%)	*p* = 0.306
Anxiety present	8 (15.7%)	11 (50.0%)		9 (34.6%)	10 (23.3%)	
Displacement from the disaster						
Did not leave the island	10 (62.5%)	9 (100.0%)	*p* = 0.035*	9 (90.0%)	10 (76.9%)	*p* = 0.412
Left the island	6 (37.5%)	0 (0.0%)		1 (10.0%)	3 (23.1%)	
Digital health literacy						
	8.02 (sd = 3.62)	8.00 (sd = 3.46)	*p* = 0.982	7.96 (sd = 3.07)	7.96 (sd = 3.83)	*p* = 0.993

^*^Significant at *p* ≤ 0.05.

^**^Significant at *p* ≤ 0.01.

### 3.2 Qualitative results

The research team conducted semi-structured in-depth interviews with 15 patients at the FQHC. All interviews were conducted in English. All participants were female (*n* = 15) and the average age was 49.1 years old (sd = 18.1). The main NCD diagnoses were anxiety and/or depression (40.0%, *n* = 6), hypercholesterolemia (33.3%, *n* = 5), metabolic diseases (diabetes mellitus type 2, pre-diabetes, borderline diabetes) (26.7%, *n* = 4), respiratory diseases (asthma or COPD) (26.7%, *n* = 4), cardiovascular diseases (arrhythmia or CHF) (30.0%, *n* = 3), hypertension (13.3%, *n* = 2), and other diseases that did not fall into the aforementioned categories (26.7%, *n* = 4).

Five major themes emerged: (1) NCD management not considered a priority; (2) the role of community support, (3) the impact of mental health on NCD management; (4) communication strategies, and (5) challenges in NCD management (Table 3).

**Table 3:** Description of main themes.

Main theme	Description
NCD management is not a priority in the aftermath of a disaster	The NCD of participants is not always the biggest priority in the aftermath of disasters. Often participants must navigate other challenges that they believe are of greater importance than their NCD.
The importance of family and community support in addressing physical and emotional needs	Participants perceived support systems in different ways in the aftermath of disasters. Some participants described social support as family, friends, or community members who could assist in the aftermath with more labor-intensive activities, like securing the home and cleaning up.
Mental health burden in the aftermath of a disaster as a barrier to NCD management	Participants discussed the mental health burden in the aftermath of disasters. Participants (with and without diagnosed mental health conditions) acknowledged the struggle to navigate the stress and trauma that result from the impact of the hurricanes.
*Subtheme*: Mental health support systems	Participants rely on family and friends for social support to cope with their mental health.
*Subtheme*: Mental health stigma	Mental health conversations are still highly stigmatized throughout the community. Although these conversations are regularly being had in the aftermath of disasters, there is still a tendency not to acknowledge the trauma of these storms.
*Subtheme*: Lack of providers available to address mental health	Participants addressed concerns over the dearth of services available to address mental health challenges.
Communication strategies	Participants recommended different forms of communication strategies to disseminate disaster preparedness information as it pertains to NCDs. This included using social media, text messaging, and radio and broader communication strategies.
Challenges in NCD management	Participants outlined challenges related to the management of their NCD in the setting of a disaster – access to health providers, access to healthy food, access to medication.

#### 3.2.1 Theme 1: priorities in the aftermath of disasters

Participants discussed a range of challenges they prioritized addressing in the aftermath of a disaster; personal, family, or financial matters were of greater importance after a disaster than thinking about their chronic condition.

#### 3.2.2 Primary caregivers

Participants who had others to care for discussed having to focus their attention on the needs of others in the aftermath of storms. Depending on the participant and family situation, this included parents, spouses, or children. One participant described her role as primary caregiver for her husband as a priority, “*What did I do to prepare? First of all, my husband is bedridden, so I’m his caregiver. He—at the time, he was still ambulatory. Now he’s not. But he would still need help working, so it was very hard for me alone-alone to prepare. To put on the shutters. It was very hard for me alone. I will never again sit out the hurricane alone because, uh—and now that he’s bedridden, I cannot. So, that was the hard part*” (Participant 39). Another participant described similar challenges in prioritizing caring for a newborn in the immediate aftermath of the storm: “*After the storm, it was still harder, ‘cause it, like, to get back on my feet from work and having a newborn, it was very hard. So you could barely find anything in stores due to the storms. It was hard. ‘Cause there was shortage of food, shortage of water ‘cause the supply*” (Participant 25). This participant faced multiple challenges that stemmed from having a newborn. Here, the newborn’s basic necessities preceded the ability to focus on her own health and, therefore, caused greater challenges in accessing food and water.

##### 3.2.2.1 Food and housing insecurity

Participants referenced difficulties with food and housing and the urgency to address these basic necessities. One participant alluded to this, “*I wish when we had the hurricane, I wish I had lights and water. That’s what I wish I had because we did not have that. We had to go, like, to assistance or water truck to get water. But I hope—and I do not want a hurricane ever again—that we have water and light, you know, ‘cause do not have water and lights, ooh, it’s rough*” (Participant 17).

#### 3.2.3 Theme 2: mental health burden

A majority of participants referenced *some* form of mental health challenges that ensued in the aftermath of the disaster. One participant described how more recent storms reminded her of the traumas of the 2017 hurricanes, “*I realize that I’m still, you know, maybe I need to talk about it, get it out of my system because with the rain, you know, it kind of brought back, um, the aftermath of and, um, what I went through.…. Yeah, and maybe I did need to talk about it*” (Participant 22). This participant’s trauma inflicted by the impact of these storms was present many years later, reflecting the mental health burden of the disasters in the acute phase but also chronically.

##### 3.2.3.1 Mental health support systems

Participants highlighted how they relied on family and friends to better cope with their mental health challenges. These sentiments included both participants with and without a diagnosed mental health NCD. Participants acknowledged the struggle to navigate stress and trauma resulting from the storms’ impact. One participant described how they preferred to discuss these struggles with siblings, rather than seek out the support of a clinician, “*Yeah, ‘cause my—some of my sisters and I are close in age, so we just lean on one another to relieve, and so, yeah… They-they [others in the community] will more talk about it with a family and friend versus a doctor. ‘Cause your doctor—some of them will look at you crazy, like, “Mmm.”*” (Participant 25). This participant believed she could not discuss these challenges with her doctor because of a perceived discrimination by her doctor, instead electing to choose family to discuss these matters.

Another participant also found the ability to confide in her own community after realizing she was not the only one experiencing these challenges with her mental health, “*Um, well it-it was very helpful to know that I wasn’t the only one feeling-having these feelings. That majority, if not all of us, were having these feelings. Um, so it-it was just helpful to know that I was not alone with this, that it wasn’t just me. ‘Cause I tend to think, “Is it me? Is it really just me?” when it comes to my feelings, so.*” (Participant 12).

##### 3.2.3.2 Mental health stigma

Despite the above recognition of mental health challenges by participants, they still acknowledged that conversations pertaining to mental health stress and trauma are still highly stigmatized in some parts of the community. One participant described how these conversations may occur: “*Um, but I think, uh, they talk about it, but not in the same terms of saying “mental health.” In other words, they are—they are talking about the stress of it, but not putting the name “mental” on it, um, because, um, well, in our island culture, uh, there is a lot of stigmatization in terms of persons who are mentally ill. And so, uh, being labeled becomes, uh, very difficult for people to-to want to identify in that manner*” (Participant 60). The stress that is prevalent is acknowledged, but due to the sensitive nature of the topic and surrounding stigma, it is not labeled with the common phrases surrounding mental health. Participants acknowledged the need to normalize these conversations and dismantle the stigma, highlighting the need to make people more aware of their mental health: “*There’s actually people, um, that might actually agree and wanna do it. Some people might be hesitant, but awareness is one of the biggest things that, um, needs to be broadcast a little bit more. Preparedness and awareness*” (Participant 75).

##### 3.2.3.3 Lack of providers available to address mental health concerns

Participants discussed the scarcity of mental health services available to the community in the setting of a disaster. One participant highlighted an active NGO and the role they played, but still acknowledged greater challenges remain: “*So, uh, the mental health consequences are high and the need for persons to be able to help them process that is very high. But like I said, the [NGO’s] role is limited, which means that our community, unfortunately, which does not have a lot of mental health resources, have not been adequately able to respond to the needs that the community has*” (Participant 60).

#### 3.2.4 Theme 3: the role of family and community support

Different participants perceived social and community support mechanisms differently in the aftermath of the storms. For some participants, social support was focused on the physical activities that family and friends could assist with. This included more labor-intensive jobs, like securing shutters for the home or assisting in cleaning up in the aftermath. One participant described how her family supported her in managing her chronic joint pains through constant support and aid, “*Oh, they went and got a lot of ice and, you know, support on my knees and stuff like that. But, yeah, the ice was helpin’ a little bit. Mm-hmm. Then they put the warm cloth, yeah. They got the warm bag cloth on my knee, so my knee could stop hurt, and they was helping me with water and the different stuff. But it was a good help, yeah”* (Participant 17). Here, the support this participant needed was focused on her chronic pain and disability. Other participants described support as their family and friends offering up their homes when their own home was damaged or destroyed in the storm.

#### 3.2.5 Theme 4: communication strategies

To improve information dissemination related to disaster preparedness for individuals with NCDs, participants recommended using different forms of communication to reach different age groups. This included social media, more traditional text messaging, and radio communication. Although our participants’ age skewed older (average was 49.1, standard deviation = 18.1), younger participants recommended using text messages and social media, including YouTube, Facebook, and Twitter, to advertise messages targeting patients with NCDs. One participant acknowledged the role social media can play in messaging dissemination given the current age of social media: “*Well, with the way things are now, not—there’s not too much people readin’ any pamphlets, brochures or anything, so—and most people are always on social media, so I think them doin’ live broadcasts or videos, video interviews or what’s not, will be better—um, would reach the community better*” (Participant 75). Most participants felt that pamphlets were not an effective messaging strategy because more people today are active online and can easily be reached by social media.

Other participants acknowledged that not everyone could be reached by online platforms: “*Just kind of get it the best that you can. Ain’t everybody have internet; ain’t everybody have social media. So wherever is the best fit for anyone to communicate, just use the best fit for you*” (Participant 25). Other strategies included using radios, particularly to reach older adults populations that listen more frequently to radio shows. As one participant acknowledged regarding mental health outreach, “*A lot of, um, people on the island, especially the elderly, they listen to their rad-the local radio station. And they get a lot of information from-from-from there. They stay tuned to that radio. And, um, I think it’s very, very much needed for them*” (Participant 22).

#### 3.2.6 Theme 5: challenges in NCD management

Participants highlighted several challenges in effectively and appropriately managing their NCD in the aftermath of a disaster. Some participants struggled to manage their NCDs due to the limited medical services available to maintain ongoing care. As one mother described [who had asthma as well as her son], “*My-my son and I was, but like I said, it was very limited in the store [medication for asthma], ‘cause everything get damaged. So whatever I had at home, that’s what-what we had to survive on… most of it went to him, and he was a child, so*” (Participant 25). Here, this participant emphasizes the challenge of having to ration medication and prioritize her son’s NCD over her own.

Other participants affirmed losing medication when their homes were destroyed and having to navigate the limited supplies that the community had of medicines for patients. As one participant described the infrastructure available in the community to support patients’ medical needs: “*… there were many instances where persons did not have medicines, and our pharmacists were not functional in the immediate aftermath. And so those persons had difficulty getting the medications that they needed*” (Participant 60). Delays in medication delivery and distribution led to increased challenges for patients finding the necessary medication to maintain their NCDs and overall well-being. This was further limited by the inability to access healthcare services and healthcare due to destruction, inaccessible roadways, and loss of providers.

### 3.3 Data integration

Integrating quantitative and qualitative data through a narrative approach provides greater insight into the challenges and opportunities of managing NCDs in disasters, especially in the USVI. From the study, there are 4 main summary points highlighted through integration:

1. The quantitative survey identified a large proportion of individuals (39.0%, *n* = 32) with inadequate levels of preparedness for a disaster. The qualitative work provides an understanding that disseminating preparedness information to fill this gap requires a multi-pronged approach with different communication strategies to ensure they reach different age groups.
2. The quantitative survey indicated that a large proportion of patients (39.7%, *n* = 23) had not received any information about preparedness specific to their NCD and over half of patients reported that their provider had not discussed this with them (52.5%, *n* = 22). Our qualitative work indicated that this was likely because NCD management is not the first thought on people’s minds when a disaster hits and, therefore, is not usually discussed in the context of preparedness.
3. Quantitative results indicated that a significant proportion of participants had mental health problems (3.5% (*n* = 4) PTSD, 28.0% (*n* = 21) anxiety, 27.8% (*n* = 20) depression). The qualitative findings showed this was in part due to the experiences of hurricanes Irma and Maria and the lack of access to mental health services. This is further hindered by the stigma that surrounds mental health, making it more difficult to seek assistance. However, the role of family and community as forms of support is critical to inform future strategies to address mental health needs.
4. The quantitative survey identified that the people with the greatest challenge in managing their NCDs in disasters included those with more than one chronic disease and those on medication. The qualitative data triangulated this finding by highlighting that some of the greatest challenges faced during the disaster included access to medications, access to healthy food, and healthcare services. These services are often more critical for persons with multiple chronic conditions.

## 4 Discussion

Using a mixed methods approach, our study provides a thorough overview of the challenges faced by persons living with NCDs in the face of disasters and their current levels of disaster preparedness. Our quantitative data indicate that a large proportion of participants had challenges managing their NCDs due mainly to difficulty accessing medication and healthcare services. We identified several factors associated with challenges in NCD management during disasters including mental health problems, having multiple NCDs, and having a usual source of care. These findings are consistent with other literature on chronic diseases post-hurricanes Irma and Maria in the US territories that similarly highlights the high incidence of mental health problems and issues with access to medication and health care services (36). Despite recent experiences with disasters, nearly half of the participants in our study had a low level of preparedness. Persistent gaps in disaster preparedness were clarified and explained through the qualitative findings. By integrating our quantitative and qualitative results we start to understand the unmet needs of persons living with NCDs in a disaster setting including effective and timely communication, education and awareness of NCD management in a disaster, addressing the current mental health burden and its exacerbation in a disaster, and the specific disaster needs of persons with NCDs including medication, healthy food, and water. Addressing these gaps is critical to strengthening the adaptive capacity of this vulnerable population to reduce the impacts of climate-related disasters on their health and well-being.

One of the key findings in our work is the inadequate level of preparedness, as found in the quantitative survey (39% of patients had low preparedness). This finding is similar to work done in Puerto Rico, where Joshipura et al. found that 59% were not prepared for the hurricanes in Puerto Rico, with low preparedness being associated with worsened health outcomes (37). The qualitative results provided insight on how best to fill the gap through improved communication. Participants emphasized the importance of using different communication strategies to reach all age groups, as there is not a “one-size-fits-all” solution to communicate specific emergency preparedness tips to individuals living with NCDs in the USVI. Leveraging various platforms (i.e., radio, social media, TV, brochures at healthcare facilities and community centers) is essential moving forward. Other studies highlight the need to strengthen disaster preparedness plans pre-disaster, ideally through various modalities, to ensure information is better streamlined (38).

A well-known challenge for preparedness during and post-disaster for this population revolves around knowledge of how to manage NCDs during extreme weather events (39). This study highlighted that a significant portion of patients (approximately 40%) did not receive information about disaster preparedness specific to their NCD, and over half did not have their provider discuss this with them. Interviews noted this gap exists, possibly since NCD management is secondary to other necessities post-disaster, like shelter, food, and water. There is a paucity of literature providing a quantitative causal link between provider preparedness conversations with patients and improved health outcomes. However, in theory, facilitating these conversations has the potential to mitigate chronic disease exacerbations, including mental health consequences, as repeated conversations boost community resilience to extreme weather events (40). Encouraging healthcare providers to facilitate conversations requires additional support, including additional training, emergency preparedness-specific tools, capacity, and opportunity (41).

Both qualitative and quantitative methods found a significant mental health burden among those living with NCDs who had experienced Hurricanes Irma and Maria. Individual interviews discovered influencing factors contributing to the significant burden, including stigma surrounding mental health and issues with access. The impact of extreme weather events, like hurricanes, on mental health is well-studied, primarily in the US mainland, with significant associations between disasters and detrimental mental health symptoms (42). Yet, the interviews highlight a protective factor within this population, specifically the role of family and community support. Communities in Puerto Rico were found to have a similar protective factor, emphasizing the importance of pride in one’s community, trust, and communication (43). This paper also highlights the importance of addressing mental health needs today, in the absence of a disaster, in addition to providing tools to manage heightened anxiety and distress that come with an extreme weather event. Other papers have outlined the mental health burden that follows hurricanes, both in the short and the long term (44–47). We add to this literature by highlighting the persistence of these mental health impacts in the USVI and the importance of addressing them to enable affected persons to manage their other NCDs when disasters hit. Strategies to strengthen the capacity to address NCDs in disasters must include a mental health component.

In addition, our study found that among people living with NCDs, those on multiple medications and have more than one chronic illness are the most vulnerable. Our qualitative data highlighted the increased needs of this population in a disaster setting that are often left unmet – access to refrigeration for medication, healthy food, clean water, and healthcare providers. These are similar to the needs identified by Andrade et al.’s qualitative analysis of 10 communities in Puerto Rico, where they found that NCD management was complicated post-hurricanes due to healthcare access, supply chain issues, rising mental health challenges, and fuel outages leading to exacerbations (36). Disaster preparedness and response operations must integrate NCDs into all sectors. Disaster preparedness and response campaigns generally focus on the needs of food, shelter, electricity, and water but seem to lack a specific emphasis on what additional considerations are required for persons with NCDs such as diabetes, cardiovascular disease, asthma, and mental health conditions. This includes not only medication but also the ability to use electrical equipment and keep medication refrigerated. It also includes ensuring that food options dispersed during the disaster are health-conscious, particularly for people living with NCDs; many foods provided during disasters are notoriously high in sugar and salt content, which can exacerbate existing chronic conditions and lead to worsened outcomes (48).

Some of the main findings in this study are corroborated by studies in Puerto Rico that outlined the immense and neglected needs of persons living with NCDs. These included challenges accessing medication, disrupted health care delivery, unhealthy behavior due to environmental restrictions, and the role of stress and mental health (36, 49–52). Our study differs from those in Puerto Rico in that we seek to understand how prior experiences with disasters and challenges have shaped current disaster preparedness. This allows us to gain insight into how to shape strategies to strengthen the capacity to adapt to future disasters.

This work calls for strategies to integrate NCDs and disaster response that are relevant beyond the USVI and Puerto Rico. For all small island developing states (SIDS) in the Caribbean and the Pacific region that face this new double burden of climate change and NCDs, it is imperative to include NCDs in disaster planning and response. Recent reports have highlighted the interdependence of NCDs (including mental health) and climate change in SIDS (53, 54). These 37 UN member countries across regions share economic, environmental, and social vulnerabilities that require a unique approach to address needs in the face of climate change and climate-related disasters. This work in the USVI provides important information to inform strategies to start to strengthen adaptive capacity of island states and nations to reduce morbidity and mortality in the face of these climate stressors. This study lays the groundwork for how other communities can approach various gaps in emergency preparedness for persons living with NCDs while considering adaptive capacity.

We consolidate the findings of this paper to provide recommended strategies that can reduce the impact of climate-related extreme weather events on persons living with NCDs in the setting of disasters in the USVI:

1. Increase knowledge and awareness around the importance of addressing NCDs in the acute aftermath of a disaster at the community and individual levels. Multi-modal educational material can be used by providers to counsel patients with chronic diseases. Educational material can also be disseminated through traditional radio, print, and social media to ensure all generations are aware of NCD management’s importance for themselves, neighbors, and family members.
2. Develop approaches to address urgent mental health needs in a disaster setting. Campaigns to destigmatize mental health to allow for conversation, safe space to ask for help, and opportunities to offer help are needed. This should be paired with initiatives to equip lay persons with the skills to manage the immediate stress and trauma of an event, given the paucity of mental health providers in the acute setting.
3. Develop strategies to ensure access to chronic disease medication. At the health system and health facility level, predefined access points for individuals who have lost medication during a disaster must be determined. These can be within shelters or set up post-disaster at defined locations that persons with NCDs are aware of ahead of time. In addition, providing refrigeration for medication that needs cold storage.
4. Develop strategies to ensure access to healthy food during a disaster. Developing an approach to store perishable items with greater nutritional value (refrigeration through resilient energy sources) and identifying nonperishable items with lower salt content and a lower glycemic index to recommend for preparing for a disaster.

This study has a few limitations to note. Firstly, the sampling strategy leads to the possibility of selection bias, given that individuals agreed to do the survey and subsequently participated in the in-depth interviews. Similarly, the disproportionate number of females in the study and qualitative interviews means that there may have been additional perspectives from self-identified men that may have been missed. Secondly, this work is focused on patients at an FQHC and may not reflect the barriers or facilitators for persons accessing care in private clinics. However, patients at the FQHC are among the most vulnerable, ensuring that the information gathered can address the needs of those with fewer resources. Lastly, sample sizes limited statistical analysis, including regression analyses, suggesting that follow-up research should be done with larger sample sizes to specify findings.

## 5 Conclusion

This study leverages the knowledge and experience of the inhabitants of St. Thomas, who survived hurricanes Irma and Maria, to inform strategies to reduce morbidity and mortality due to poorly controlled NCDs in disasters in the future. We identified that the people who struggled most with managing their NCDs were those who had multiple chronic conditions linking this finding to heightened issues with access to healthcare, medications, and healthy food. Many participants were found to have low levels of disaster preparedness, exacerbated by a paucity of information about preparedness for their NCDs. Participants suffered from mental health problems that were exacerbated by access to mental health services and stigma. There is an urgent need to integrate NCDs and disaster response by increasing awareness around the management of NCDs in the acute aftermath of a disaster, addressing the mental health needs of survivors, and ensuring access to medication and healthy food.

## Data Availability

The raw data supporting the conclusions of this article will be made available by the authors, without undue reservation.
